# Loss of function mutations in PTPN6 promote STAT3 deregulation *via* JAK3 kinase in diffuse large B-cell lymphoma

**DOI:** 10.18632/oncotarget.6300

**Published:** 2015-11-09

**Authors:** Christos Demosthenous, Jing Jing Han, Guangzhen Hu, Mary Stenson, Mamta Gupta

**Affiliations:** ^1^ Division of Hematology, Department of Internal Medicine, Mayo Clinic, College of Medicine, Rochester, MN, USA

**Keywords:** PTPN6 mutations, STAT3, DLBCL, JAK kinases

## Abstract

PTPN6 (SHP1) is a tyrosine phosphatase that negatively controls the activity of multiple signaling pathways including STAT signaling, however role of mutated PTPN6 is not much known. Here we investigated whether PTPN6 might also be a potential target for diffuse large B cell lymphoma (DLBCL) and performed Sanger sequencing of the PTPN6 gene. We have identified missense mutations within PTPN6 (N225K and A550V) in 5% (2/38) of DLBCL tumors. Site directed mutagenesis was performed to mutate wild type (WT) PTPN6 and stable cell lines were generated by lentiviral transduction of PTPN6^WT^, PTPN6^N225K^ and PTPN6^A550V^ constructs, and effects of WT or mutated PTPN6 on STAT3 signaling were analyzed. WT PTPN6 dephosphorylated STAT3, but had no effect on STAT1, STAT5 or STAT6 phosphorylation. Both PTPN6 mutants were unable to inhibit constitutive, as well as cytokines induced STAT3 activation. Both PTPN6 mutants also demonstrated reduced tyrosine phosphatase activity and exhibited enhanced STAT3 transactivation activity. Intriguingly, a lack of direct binding between STAT3 and WT or mutated PTPN6 was observed. However, compared to WT PTPN6, cells expressing PTPN6 mutants exhibited increased binding between JAK3 and PTPN6 suggesting a more dynamic interaction of PTPN6 with upstream regulators of STAT3. Consistent with this notion, both the mutants demonstrated increased resistance to JAK3 inhibitor, WHIP-154 relative to WT PTPN6. Overall, this is the first study, which demonstrates that N225K and A550V PTPN6 mutations cause loss-of-function leading to JAK3 mediated deregulation of STAT3 pathway and uncovers a mechanism that tumor cells can use to control PTPN6 substrate specificity.

## INTRODUCTION

PTPN6, also known as SHP1, is a ubiquitously expressed SH2 domain-containing PTP.[1–3] In humans, the PTPN6 gene is encoded by 17 exons and has 2 promoter regions.[4] PTPN6 1A (or P1) is the longer region and is expressed primarily in non-hematopoietic cells, whereas the shorter region, PTPN6 1B (or P2) is expressed only in cells of hematopoietic lineage.[1, 2, 4] PTPN6 contains two Src-homology 2 domains that allow attachment to the phospho-tyrosine residues present on signaling molecules. This interaction triggers activation of the catalytic domain and the subsequent dephosphorylation of the substrate.[5] Loss of function of PTPN6 in murine models has been previously described as a driver of autoimmune diseases.[6–8] Motheaten mice carrying the autosomal recessive motheaten (*Ptpn6^me^*) and viable motheaten (*Ptpn6^me-v^*) mutations were shown to develop immunodeficiency and several pathophysiological abnormalities.[7–9] In this study we investigated PTPN6 mutations in diffuse large B cell lymphoma and characterized their role in deregulation of STAT3 signaling pathway.

Diffuse large B-cell lymphoma (DLBCL) is the most common non-Hodgkin common lymphoma worldwide accounting for about 30% of newly diagnosed cases in the United States.[10] Although most patients achieve complete remission in response to the current frontline therapy, R-CHOP, approximately 40% of patients fail to respond to initial treatment and eventually die of disease.[11] So far, many signal transduction pathways have been implicated in the pathogenesis of DLBCL such as the BCR signaling, [12] NF-κB [13], [14] and mTOR pathways. Furthermore, high expression of STAT3 protein in DLBCL tumors as detected by immunohistochemistry (IHC) has been associated with unfavorable outcome in some, [15] but not all studies.[16–18] In addition, previous studies have demonstrated deregulation of JAK/STAT3 signaling in DLBCL.[16, 19, 20].

The mechanism of aberrant STAT3 activation in cancer is not well understood. Several potential causes have been suggested and studied including deregulated cytokine secretion (e.g. IL-10), [21] gain of function mutations, [22–24] and loss of negative regulators of JAK/STAT signaling such as SOCS1 [25–27] and protein tyrosine phosphatases.[28, 29] Protein tyrosine phosphatases (PTP) are important enzymes that negatively regulate the activity of multiple signaling pathways downstream of tyrosine kinases including the Janus kinases.[1, 30, 31] However, the role of PTPN6 mutations in the regulation of STAT signaling has not previously been described in DLBCL. The present study was designed to evaluate the functional significance of PTPN6 mutations on STAT signaling.

## RESULTS

### Identification of PTPN6 mutations in DLBCL tumors

**Figure 1:**
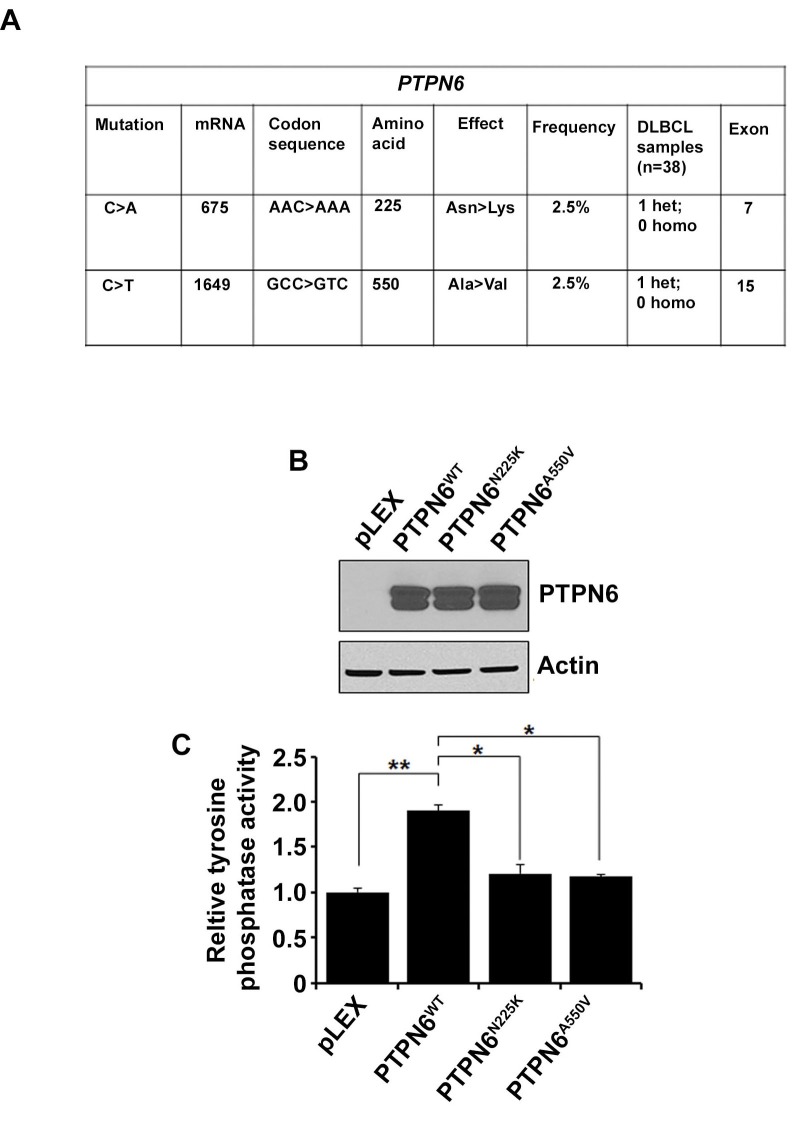
Identification of PTPN6 mutations in DLBCL tumors **A.** Data summarizing PTPN6 mutations in DLBCL tumors (*n* = 38). **B.** Western blot analysis shows overexpression of PTPN6 in stably transfected HEK293T cells by PTPN6^WT^, PTPN6^N225K^ and PTPN6^A550V^ plasmids. **C.** Protein tyrosine phosphatase assay was performed in the PTPN6 WT and mutant stably transfected cells. Bars represent mean ± SD from 3 different experiments (*P < 0.05, **P < 0.005).

To identify the PTPN6 mutations, DNA from 38 DLBCL tumor samples was sequenced bidirectionally. All exons of PTPN6 gene were amplified and analyzed by Sanger sequencing. Two novel heterozygous missense mutations were identified in 2 separate patient tumors. The first mutation was located in exon 7 and resulted in an Asparagine to Lysine substitution at codon 225 (N225K). The second missense mutation found, in exon 15, resulted in Alanine to Valine substitution at codon 550 (A550V) (Figure 1A). The locations of the N225K and A550V mutations are depicted in the modular structure of the PTPN6 gene (S1). Alignment analysis showed that the human N225 asparagine and the A550 alanine amino acids of the PTPN6 protein differ among various species (S1).

In order to elucidate the functional significance of these mutations, site directed mutagenesis of PTPN6^WT^ was performed. Lentiviral vectors pLEX-PTPN6^WT^, pLEX-PTPN6^N225K^ and pLEX-PTPN6^A550V^ were constructed, and overexpressed in HEK293T cells. PTPN6 mutations did not affect expression or stability of PTPN6 at the protein level (Figure 1B). Interestingly, both mutants demonstrated reduction of tyrosine phosphatase activity as compared to PTPN6^WT^ (Figure 1C), suggesting that these PTPN6 mutations are loss of function mutations.

### PTPN6 mutants lost the activity to dephosphorylate constitutive STAT3

**Figure 2:**
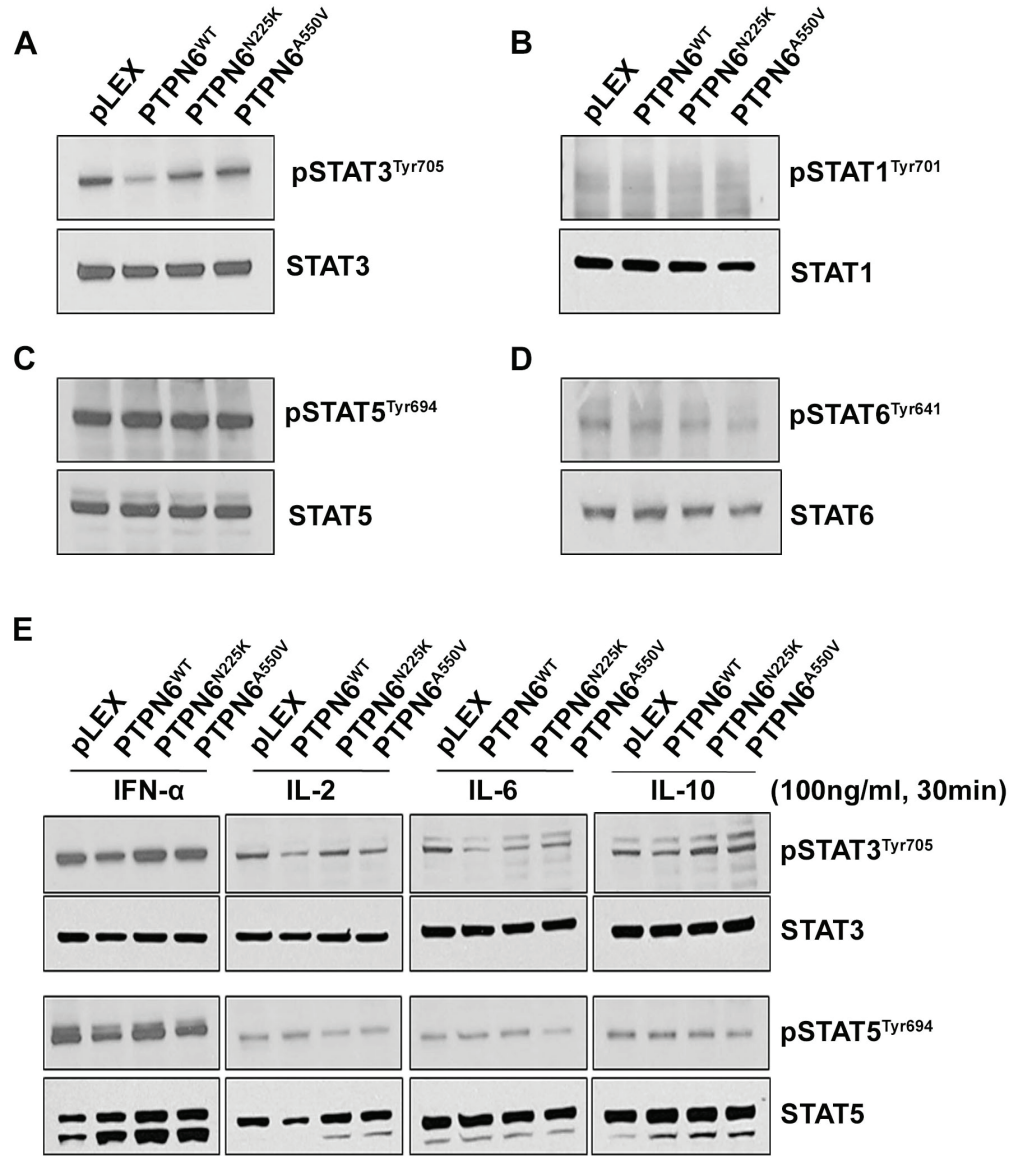
The effect of N225K and A550V PTPN6 mutations on constitutive or cytokines induced STATs phosphorylation Constitutive level of STAT3 **A.**, STAT1 **B.**, STAT5 **C.** and STAT6 **D.** tyrosine phosphorylation was assessed in PTPN6^WT^, PTPN6^N225K^ and PTPN6^A550V^ stably transfected HEK293T cells by western blotting (*n* = 3). **E.** Serum-starved transfected HEK293 cells were treated with 100 ng/mL of IFN-α, IL-2, IL-6 and IL-10 and for 30 minutes as indicated and phosphorylation of STAT3 and STAT5 were assessed by Western blot (*n* = 2).

As tyrosine phosphorylation is essential for STAT signaling and since PTPs are important negative regulators of the pathway, [32] we examined the effect of PTPN6^N225K^ and PTPN6^A550V^ mutations on STAT1, STAT3, STAT5 and STAT6 constitutive phosphorylation in stably transfected PTPN6 mutant and WT cell lines. Western blot analysis showed that while overexpression of WT PTPN6 decreased STAT3 phosphorylation as compared to cells with empty vector, cells expressing PTPN6 mutants maintained STAT3 phosphorylation comparable to the cells with transduced with empty vector (Figure 2A). Constitutive phosphorylated tyrosine levels of STAT1 (Figure 2B), STAT5 (Figure 2C) or STAT6 (Figure 2D) were similar in PTPN6 mutants and WT cells (Figure 2A–2D).

A variety of cytokines activate STAT signaling by binding to cell surface receptors triggering the activity of receptor-associated Janus kinase (JAK) family members.[33] Stimulation of cells expressing WT PTPN6 with IFN-α, IL-2, IL-6 or IL-10 resulted in decreased phosphorylation of STAT3 but not STAT5 as compared to the cells transduced with empty vector (Figure 2E). Interestingly, neither STAT3 nor STAT5 phosphorylation changed in cells expressing PTPN6 mutants in response to cytokine treatments as compared to cells transduced with empty vector (Figure 2E). Taken together these results indicate that PTPN6 mutations, N225K or A550V can deregulate STAT3 phosphorylation in cancer cells.

### Binding of PTPN6 mutants with STAT3 and its upstream activators JAK1-3 kinases

**Figure 3:**
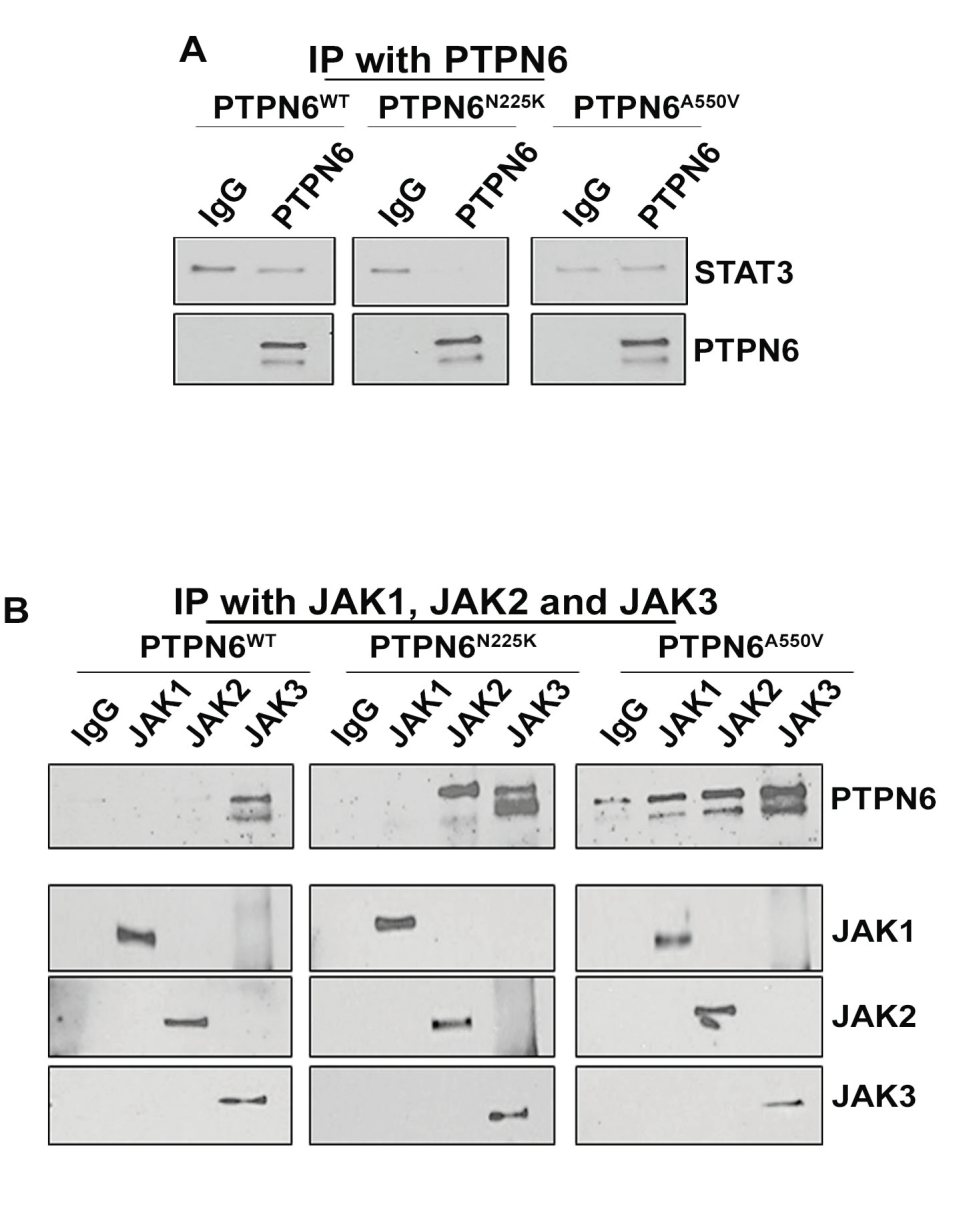
Interaction between WT and mutants PTPN6 with STAT3 or JAK kinases **A.** Immunoprecipitation was performed in HEK293^PTPN6/WT^, HEK293^PTPN6/N225K^ and HEK293^PTPN6/A550V^ cells with PTPN6 antibody, followed by immunoblotting with STAT3 antibody (*n* = 2). **B.** JAK1, JAK2 and JAK3 were immunoprecipitated from lysates of the transfected HEK293^PTPN6/WT^, HEK293^PTPN6/N225K^ and HEK293^PTPN6/A550V^ cells and the immuno-complexes were examined for presence of PTPN6 (*n* = 3).

PTPN6 acts as a negative regulator of intracellular signaling by inhibiting the recruitment of transmembrane receptors with intrinsic tyrosine kinase activity.[34] To investigate whether PTPN6 and STAT3 physically interact, we pulled down PTPN6 from HEK293T cells overexpressing PTPN6^WT^, PTPN6^N225K^ and PTPN6^A550V^ and assessed the presence of STAT3 in PTPN6 immunoprecipitates. As shown in Figure 3A, we could not detect STAT3 in PTPN6 immunoprecipitates from cells expressing either PTPN6 mutants or WT constructs (Figure 3A). These results suggest lack of direct physical interaction between PTPN6 and STAT3. While our results overruled a direct interaction between STAT3 and PTPN6, PTPN6 may potentially regulate STAT3 activity by interacting with upstream JAK kinases such as JAK1, JAK2 and JAK3. In order to detect association between PTPN6 and the JAK proteins, we co-immunoprecipitated JAK1, JAK2 and JAK3 from the cells overexpressing WT or mutant PTPN6 and assessed the presence of PTPN6. In WT PTPN6 expressing cells, PTPN6 binding was detected only in JAK3 immunoprecipitates, but not in JAK1 or JAK2 immunoprecipitates. Moreover, the PTPN6 mutants tend to bind with high affinity with JAK3 as compared to WT PTPN6, a low affinity binding of PTPN6 mutants was also observed with JAK1 or JAK2 (Figure 3B). Overall, these data demonstrate that the ability of PTPN6 to dephosphorylate STAT3 is mediated by an increased physical interaction with JAK3 kinase. Enhanced binding observed between JAK3 and PTPN6 mutants may potentially arise from lack of PTPN6 enzymatic activity required for changes in the JAK-STAT signaling complex (Figure 3B).

### Effects of pharmacological inhibition of JAK1/2 or JAK3 on STAT3 phosphorylation in cells expressing WT or mutants PTPN6

**Figure 4:**
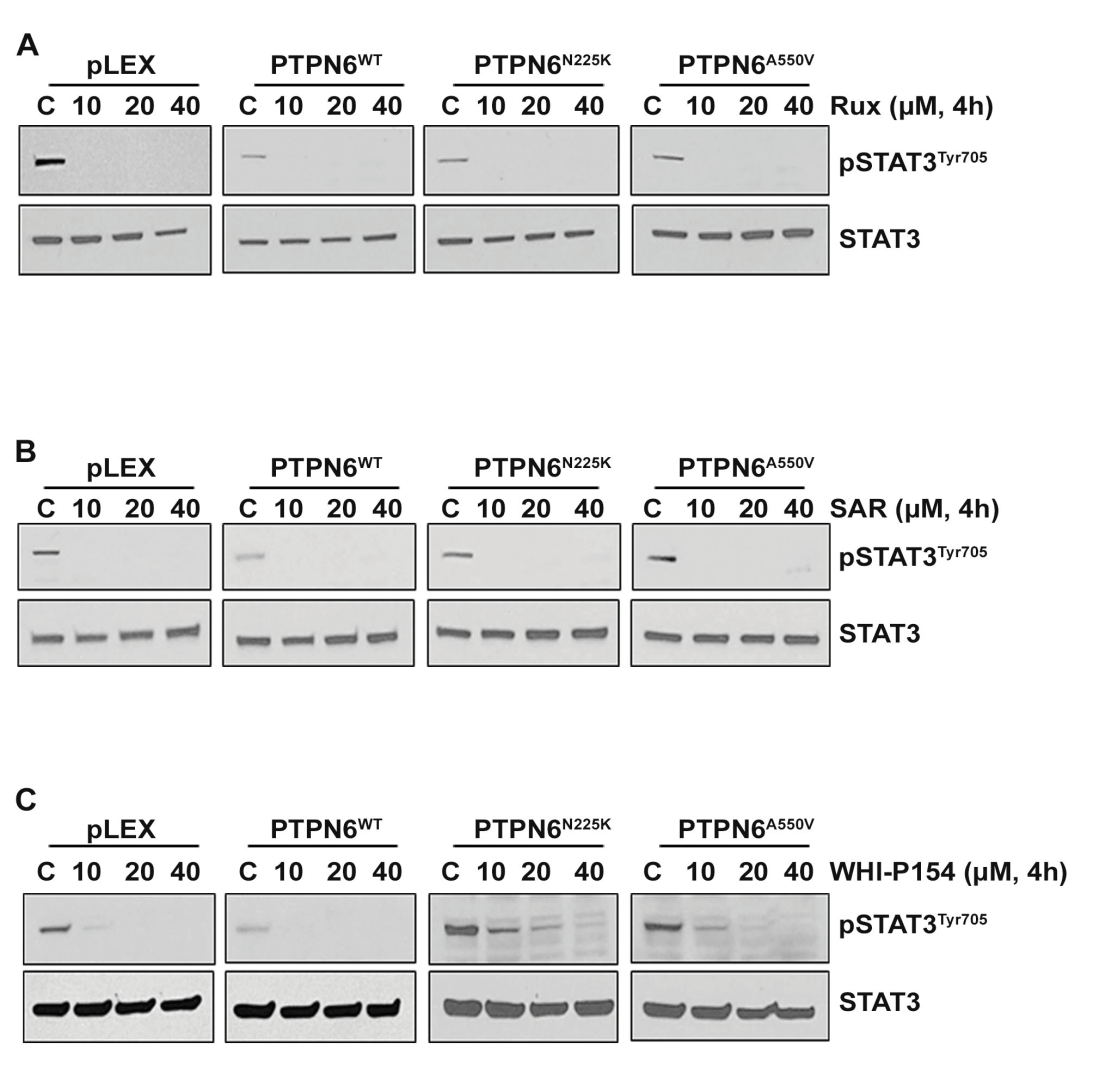
Effect of JAK kinase inhibitors on STAT3 phosphorylation in PTPN6^WT^ and mutants (PTPN6^N225K^ and PTPN6^A550V^) expressing cells **A.**-**C.** Empty vector, PTPN6^WT^, PTPN6^N225K^ and PTPN6^A550V^ over-expressing cells were treated for 4 hours with various concentrations of **A.** Ruxolitinib, **B.** SAR302503 and **C.** WHI-P154 and STAT3 phosphorylation was assessed by western blotting. Experiments were repeated 3 times with similar results.

The JAK/STAT pathway is considered a promising target in DLBCL and several other types of cancer, and many JAK kinase inhibitors are being tested in clinic. To determine whether PTPN6 modulates cellular sensitivity to pharmacological inhibitors of JAK1, JAK2 or JAK3 kinases, we treated 293T cells expressing WT or mutant PTPN6 with various concentrations of the Ruxolitinib (JAK1/JAK2 selective inhibitor), SAR302503 (a JAK2 specific inhibitor) or WHI-P154 (JAK3 specific inhibitor). Treatment with pharmacological inhibitors Ruxolitinib and SAR302503 inhibited the phosphorylation of STAT3 in cells expressing WT PTPN6 or mutants (Figure 4A–4B). Treatment with the JAK3 inhibitor WHI-P154 completely suppressed STAT3 phosphorylation only in cells expressing WT PTPN6 cells at all the various concentrations tested. In contrast, a dose dependent effect of WHI-P154 on STAT3 phosphorylation was observed in cells expressing PTPN6 mutants, and complete dephosphorylation was observed at 3-5 fold higher concentrations of WHI-P154, suggesting that loss of PTPN6 activity due to mutations may cause resistance to the JAK3 inhibition (Figure 4C).

### PTPN6 modulated STAT3 mediated gene expression

**Figure 5:**
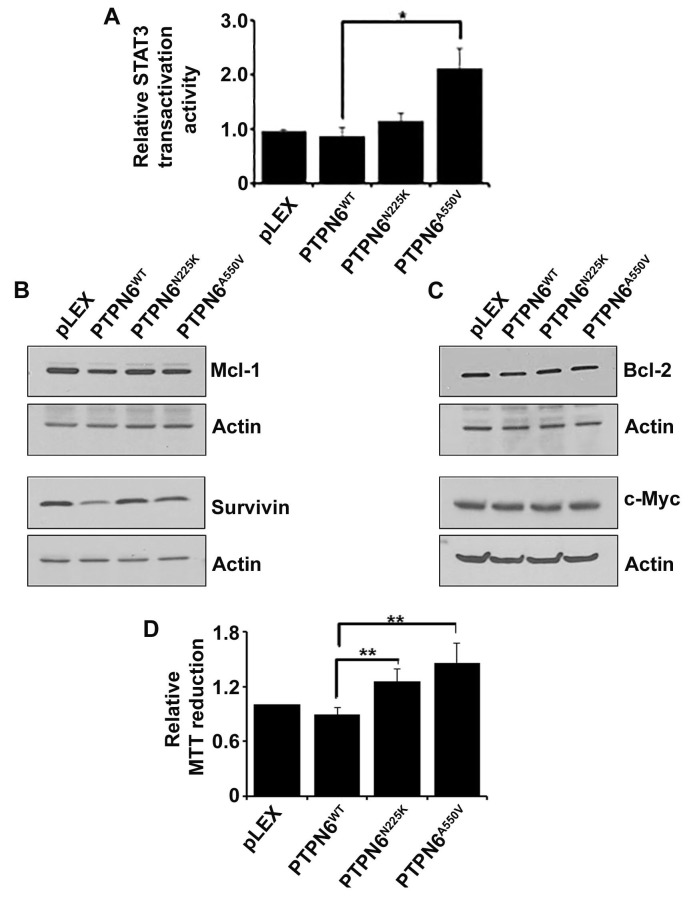
The effect of PTPN6^WT^, PTPN6^N225K^ and PTPN6^A550V^ on STAT3 transactivation activity, STAT3 direct downstream targets and cell proliferation **A.** STAT3 transactivation activity was evaluated by luciferase reporter assay in the HEK293T stably transfected cells. Briefly the HEK293T were transiently transfected with a STAT3 luciferase reporter and luciferase assay was performed in empty vector, PTPN6^WT^ and mutants PTPN6^N225K^ and PTPN6^A550V^. Bars represent mean ± SD from 3 different experiments (*P < 0.05). **B.**-**C.** Effect of PTPN6^WT^, PTPN6^N225K^ and PTPN6^A550V^ on STAT3 direct targets, Mcl-1 and survivin **B.**; Bcl-2 and c-Myc **C.** were evaluated by western blotting (*n* = 3). **D.** MTT assay was performed in the HEK293T stably transfected cells as the readout for cell proliferation. Bars represent mean ± SD from 3 different experiments (**P < 0.005).

To examine whether PTPN6 can influence STAT3 transactivation activity, WT or mutant PTPN6 constructs were cotransfected with a STAT3-driven luciferase reporter in to HEK293T cells and luciferase assays were carried out. As demonstrated in Figure 5A, compared to WT PTPN6, which significantly decreased transactivation activity of STAT3, PTPN6 mutants had only moderate decline in STAT3 transactivation activity, which is relatively more evident in cells transfected with PTPN6^A550V^ cells (Figure 5A).

*Bcl-2*, survivin, *Mcl-1* and *c-Myc* are important downstream targets of STAT3.[35] We sought to determine whether PTPN6 mutations could affect expression of these STAT3 targets. Overexpression of WT PTPN6 inhibited the protein levels of Mcl-1 and survivin but no effect on Bcl-2 and c-Myc (Figure 5B–5C). On the other hand Mcl-1 and survivin levels were not decreased in PTPN6 mutants overexpressing cells (Figure 5B–5C). Additionally, overexpression of WT PTPN6 slightly protected against cell proliferation as compared to the vector alone, however overexpression of PTPN6 mutants promoted cell proliferation (Figure 5D). Taken together these results indicate that *via* disrupting phosphatase activity of PTPN6, genetic mutations in *PTPN6* potentiate oncogenic effects of STAT3.

## DISCUSSION

To further improve the clinical outcomes in DLBCL, current research is aimed on increasing the understanding of the pathophysiology of this disease by focusing on the study of the molecular mechanisms involved in the pathogenesis of lymphoma. STAT3 deregulation is shown to be present in 35-40% of DLBCL cases.[16, 36] The approval of the JAK1/JAK2 inhibitor Ruxolitinib in patients with myeloproliferative neoplasms [37] has increased investigations as to whether inhibiting JAK/STAT signaling will be effective in DLBCL. Herein, we demonstrate that loss-of-function mutations in protein tyrosine phosphatase PTPN6, a negative regulator of JAK/STAT pathway, [3, 30, 38, 39] leads to deregulated STAT3 signaling and accumulation of proto-oncogenes that are direct downstream targets of STAT3. Epigenetic silencing of PTPN6, either due to DNA methylation or histone modification, as a contributive factor for deregulated JAK/STAT signaling has been previously described in various hematologic malignancies.[27, 28, 40] However, the role of PTPN6 mutations in DLBCL remains unclear. In the present study, we found 2 heterozygous missense mutations in the PTPN6 gene. N225K and A550V mutations were identified in 2 separate patient tumors from a set of 38 DLBCL tumors (2/38; 5.2%), indicating that PTPN6 mutations in DLBCL tumors are uncommon.

Interestingly, both mutations demonstrated decreased tyrosine phosphatase activity as compared to WT PTPN6, suggesting that both mutations are loss of function mutations. The presence of decreased phosphatase activity in PTPN6 mutations raised the question of whether those mutations play a role in JAK/STAT3 deregulation. Consistent with previous reports, PTPN6^WT^ was able to dephosphorylate basal level of tyrosine of STAT3, [40–42] while N225K and A550V mutations were shown to decrease the ability of PTPN6 to suppress constitutive pSTAT3 levels. The same pattern was observed after IFN-α, IL-2, IL-6 and IL-10 stimulation. A study recently demonstrated the important role of PTPN6 as a negative regulator of STAT3 phosphorylation in response to cytokine stimulation with IL-6. The same study, also demonstrated the decreased ability of PTPN6 to dephosphorylate STAT3 in moth-eaten mice (me/+ and me/me phenotypes).[42] Although PTPN6 was also shown to inactivate the JAK/STAT pathway by dephosphorylation of the JAK kinases, [43] the physical association between STAT3 and PTPN6 has not been illustrated before. In view of the tyrosine phosphatase property of PTPN6, a possible additional mechanism through which PTPN6 dephosphorylates STAT3 is direct inactivation using STAT proteins as substrates. Our findings, however, suggest that binding between PTPN6 and STAT3 does not exist and these data come in contrast with a previous hypothesis by Paling et al. [44] Han et al. [45] demonstrated that loss of PTPN6 enhances JAK3/STAT3 interaction in ALK + anaplastic large-cell lymphoma. By performing immunoprecipitation studies, we found that direct interaction of JAK3 with PTPN6 was enhanced in PTPN6 mutants as compared to WT PTPN6. Our finding is consistent with recent study demonstrated that PTPN6 has increased affinity towards phosphorylated JAK kinases.[46] Taken together with our data, we propose that PTPN6 regulates STAT3 signaling through deactivation of the upstream modulators such as JAK3 kinase.

The proto-oncogenic role of constitutively active STAT3 in tumor growth and invasion occurs due to transcription of genes related to tumor cell survival.[47, 48] Persistent activation of STAT3 in tumor cells participates in the expression and regulation of genes involved in controlling apoptosis such as Mcl-1, Bcl-2, survivin and c-Myc.[35, 49] The inhibitory effect of PTPN6^WT^ on Mcl-1, Bcl-2 and survivin is already known.[45] Consistently, our results imply that while overexpression of PTPN6^WT^ via inhibition of STAT3 suppresses levels of Mcl-1 and survivin, mutant PTPN6 results in accumulation of these proteins. The promising results of Ruxolitinib in patients with myelofibrosis and the emerging relevance of the JAK-STAT pathway in DLBCL pathogenesis, has led to an ongoing clinical trial in patients with relapsed DLBCL and T-cell lymphoma (Clinical Trials.gov Identifier: NCT01431209). In the studies reported here, we sought to address the question whether PTPN6 mutations affect response to Ruxolitinib and other JAK kinase inhibitors. Of importance, and contrary to WT PTPN6, PTPN6 mutants were slightly resistant to JAK3 inhibition. In summary, this study provides evidence that N225K and A550V mutations in PTPN6 are able to cause loss-of-function of PTPN6 leading to increased binding to JAK3 kinase and resulted in STAT3 deregulation and accumulation of survivin and Mcl-1.

## MATERIALS AND METHODS

### Cell lines

HEK293T cell line was from Open Biosystem (Huntsville, AL, USA) and was grown in the Dulbecco's Modified Eagle Medium supplemented with 10% Fetal Bovine Serum.

### Antibodies and reagents

Phospho-specific antibodies to STAT1^Tyr701^, STAT3^Tyr705^, STAT5^Tyr694^ and STAT6^Tyr641^ were from Cell Signaling Technologies (Beverly, MA, USA). STAT1, STAT3, STAT5, STAT6, JAK1, JAK2, JAK3, PTPN6, Bcl-2, c-Myc, Mcl-1 and survivin antibodies were also from Cell Signaling Technologies. Actin antibody was purchased from Santa Cruz (Santa Cruz, CA, USA). Recombinant human IL-2, IL-6 and IL-10 were from Peprotech (Rocky Hill, NJ, USA). IFN-α was purchased from Sigma Aldrich (St. Louis, MO, USA).

### Drugs

Ruxolitinib (RUX) was purchased from ChemieTek (Indianapolis, IN, USA) and WHI-P154 was from Santa Cruz (Dallas, TX, USA). TG101348 (TG) was a gift from TargeGEN Pharmaceuticals (now Sanofi-Aventis) (San Diego, CA, USA). TG has now been renamed SAR302503 (SAR).

### Identification of PTPN6 mutations in DLBCL tumors

For PTPN6 sequencing DNA was used from DLBCL tumors (*n* = 38) as previously described.[22, 50] PCR amplification of the PTPN6 gene (all the 17 exons) was performed and the PCR fragments were sequenced and analyzed at the Mayo Clinic Cancer Center Gene Analysis Core Facility.

### Site directed mutagenesis to create PTPN6 mutants

The coding region of PTPN6 (NM_002831.5) was amplified and cloned into vector TOPO TA (Invitrogen, Grand Island, NY, US). The plasmids of PTPN6 with 675C > A and 1649C > T mutations in the coding region of PTPN6 were created by site-directed mutagenesis as described.[22]

### Lentiviral transduction

The cells overexpressing PTPN6^WT^, PTPN6^N225K^ and PTPN6^A550V^ were created by transducing cells with lentiviral constructs as described previously.[22]

### Tyrosine phosphatase activity assay

Phosphatase activity of PTPN6 was assessed using the RediPlate 96 EnzChek Tyrosine Phosphatase Assay kit (Molecular Probes, Eugene, OR, USA). After an overnight serum starvation, cells were washed with saline before lysis in buffer containing 50mM MOPS, 50mM NaCl, 1mM DTT and 0.3% Tween and protease inhibitor. Lysates were transferred into RediPlate wells and incubated for 30 min at 20-22° C before reading for fluorescence.

### Western blotting and co-immunoprecipitation

Western blotting was performed as described earlier.[51] For the co-immunoprecipitation assay, 5 μg of specific antibodies were added to the lysates and incubated with rotation at 4°C to allow complex formation. 30 μl of protein G-Agarose was then added and the incubation continued overnight. Immunoprecipitates were captured with Agarose beads; washed four times with RIPA buffer; and analyzed by western blotting.

### Luciferase assay

The effect of PTPN6^WT^, PTPN6^N225K^ and PTPN6^A550V^ on STAT3 transactivation activity was measured by a luciferase assay as described before.[22]

### Cell proliferation assay

Cells were seeded in 96 well plates and incubated at 37°C. MTT 3-(4,5-dimethylthiazol-2-yl)-2,5-diphenyltetrazolium was added, followed by incubation at 37°C for 4 hours. After addition of isopropanol containing 0.04N HCL, absorbance readings were taken at a wavelength of 570nm using spectrophotometer.

### Statistics

The *p*-values for *in-vitro* data were calculated using the means from 3 different experiments (two-tailed unpaired Student's t test).

## SUPPLEMENTARY MATERIAL FIGURE



## References

[1] Wu C, Sun M, Liu L, Zhou GW (2003). The function of the protein tyrosine phosphatase SHP-1 in cancer. Gene.

[2] Paling NR, Welham MJ (2005). Tyrosine phosphatase SHP-1 acts at different stages of development to regulate hematopoiesis. Blood.

[3] Tsui FW, Martin A, Wang J, Tsui HW (2006). Investigations into the regulation and function of the SH2 domain-containing protein-tyrosine phosphatase, SHP-1. Immunologic research.

[4] Banville D, Stocco R, Shen SH (1995). Human protein tyrosine phosphatase 1C (PTPN6) gene structure: alternate promoter usage and exon skipping generate multiple transcripts. Genomics.

[5] Valentino L, Pierre J (2006). JAK/STAT signal transduction: regulators and implication in hematological malignancies. Biochemical pharmacology.

[6] Croker BA, Lawson BR, Rutschmann S, Berger M, Eidenschenk C, Blasius AL, Moresco EM, Sovath S, Cengia L, Shultz LD, Theofilopoulos AN, Pettersson S, Beutler BA (2008). Inflammation and autoimmunity caused by a SHP1 mutation depend on IL-1, MyD88, and a microbial trigger. Proceedings of the National Academy of Sciences of the United States of America.

[7] Nesterovitch AB, Szanto S, Gonda A, Bardos T, Kis-Toth K, Adarichev VA, Olasz K, Ghassemi-Najad S, Hoffman MD, Tharp MD, Mikecz K, Glant TT (2011). Spontaneous insertion of a b2 element in the ptpn6 gene drives a systemic autoinflammatory disease in mice resembling neutrophilic dermatosis in humans. The American journal of pathology.

[8] Shultz LD, Rajan TV, Greiner DL (1997). Severe defects in immunity and hematopoiesis caused by SHP-1 protein-tyrosine-phosphatase deficiency. Trends in biotechnology.

[9] Lyons BL, Smith RS, Hurd RE, Hawes NL, Burzenski LM, Nusinowitz S, Hasham MG, Chang B, Shultz LD (2006). Deficiency of SHP-1 protein-tyrosine phosphatase in “viable motheaten” mice results in retinal degeneration. Investigative ophthalmology & visual science.

[10] Siegel R, Naishadham D, Jemal A (2013). Cancer statistics, 2013. CA: a cancer journal for clinicians.

[11] Friedberg JW (2011). Relapsed/refractory diffuse large B-cell lymphoma. Hematology / the Education Program of the American Society of Hematology American Society of Hematology Education Program.

[12] Young RM, Staudt LM (2013). Targeting pathological B cell receptor signalling in lymphoid malignancies. Nature reviews Drug discovery.

[13] Davis RE, Brown KD, Siebenlist U, Staudt LM (2001). Constitutive nuclear factor kappaB activity is required for survival of activated B cell-like diffuse large B cell lymphoma cells. The Journal of experimental medicine.

[14] Lam LT, Davis RE, Pierce J, Hepperle M, Xu Y, Hottelet M, Nong Y, Wen D, Adams J, Dang L, Staudt LM (2005). Small molecule inhibitors of IkappaB kinase are selectively toxic for subgroups of diffuse large B-cell lymphoma defined by gene expression profiling. Clinical cancer research : an official journal of the American Association for Cancer Research.

[15] Wu ZL, Song YQ, Shi YF, Zhu J (2011). High nuclear expression of STAT3 is associated with unfavorable prognosis in diffuse large B-cell lymphoma. Journal of hematology & oncology.

[16] Gupta M, Maurer MJ, Wellik LE, Law ME, Han JJ, Ozsan N, Micallef IN, Dogan A, Witzig TE (2012). Expression of Myc, but not pSTAT3, is an adverse prognostic factor for diffuse large B-cell lymphoma treated with epratuzumab/R-CHOP. Blood.

[17] Gupta M, Ansell SM, Novak AJ, Kumar S, Kaufmann SH, Witzig TE (2009). Inhibition of histone deacetylase overcomes rapamycin-mediated resistance in diffuse large B-cell lymphoma by inhibiting Akt signaling through mTORC2. Blood.

[18] Majchrzak A, Witkowska M, Smolewski P (2014). Inhibition of the PI3K/Akt/mTOR signaling pathway in diffuse large B-cell lymphoma: current knowledge and clinical significance. Molecules.

[19] Lam LT, Wright G, Davis RE, Lenz G, Farinha P, Dang L, Chan JW, Rosenwald A, Gascoyne RD, Staudt LM (2008). Cooperative signaling through the signal transducer and activator of transcription 3 and nuclear factor-{kappa}B pathways in subtypes of diffuse large B-cell lymphoma. Blood.

[20] Ding BB, Yu JJ, Yu RY, Mendez LM, Shaknovich R, Zhang Y, Cattoretti G, Ye BH (2008). Constitutively activated STAT3 promotes cell proliferation and survival in the activated B-cell subtype of diffuse large B-cell lymphomas. Blood.

[21] Gupta M, Han JJ, Stenson M, Maurer M, Wellik L, Hu G, Ziesmer S, Dogan A, Witzig TE (2012). Elevated serum IL-10 levels in diffuse large B-cell lymphoma: a mechanism of aberrant JAK2 activation. Blood.

[22] Hu G, Witzig TE, Gupta M (2013). A novel missense (M206K) STAT3 mutation in diffuse large B cell lymphoma deregulates STAT3 signaling. PloS one.

[23] Morin RD, Mendez-Lago M, Mungall AJ, Goya R, Mungall KL, Corbett RD, Johnson NA, Severson TM, Chiu R, Field M, Jackman S, Krzywinski M, Scott DW, Trinh DL, Tamura-Wells J, Li S (2011). Frequent mutation of histone-modifying genes in non-Hodgkin lymphoma. Nature.

[24] Lohr JG, Stojanov P, Lawrence MS, Auclair D, Chapuy B, Sougnez C, Cruz-Gordillo P, Knoechel B, Asmann YW, Slager SL, Novak AJ, Dogan A, Ansell SM, Link BK, Zou L, Gould J (2012). Discovery and prioritization of somatic mutations in diffuse large B-cell lymphoma (DLBCL) by whole-exome sequencing. Proceedings of the National Academy of Sciences of the United States of America.

[25] Mottok A, Renne C, Seifert M, Oppermann E, Bechstein W, Hansmann ML, Kuppers R, Brauninger A (2009). Inactivating SOCS1 mutations are caused by aberrant somatic hypermutation and restricted to a subset of B-cell lymphoma entities. Blood.

[26] Schif B, Lennerz JK, Kohler CW, Bentink S, Kreuz M, Melzner I, Ritz O, Trumper L, Loeffler M, Spang R, Moller P (2013). SOCS1 mutation subtypes predict divergent outcomes in diffuse large B-Cell lymphoma (DLBCL) patients. Oncotarget.

[27] Chim CS, Wong KY, Loong F, Srivastava G (2004). SOCS1 and SHP1 hypermethylation in mantle cell lymphoma and follicular lymphoma: implications for epigenetic activation of the Jak/STAT pathway. Leukemia.

[28] Witzig TE, Hu G, Offer SM, Wellik LE, Han JJ, Stenson MJ, Dogan A, Diasio RB, Gupta M (2014). Epigenetic mechanisms of protein tyrosine phosphatase 6 suppression in diffuse large B-cell lymphoma: implications for epigenetic therapy. Leukemia.

[29] Aya-Bonilla C, Camilleri E, Haupt LM, Lea R, Gandhi MK, Griffiths LR (2014). In silico analyses reveal common cellular pathways affected by loss of heterozygosity (LOH) events in the lymphomagenesis of Non-Hodgkin's lymphoma (NHL). BMC genomics.

[30] Ostman A, Hellberg C, Bohmer FD (2006). Protein-tyrosine phosphatases and cancer. Nature reviews Cancer.

[31] Xu D, Qu CK (2008). Protein tyrosine phosphatases in the JAK/STAT pathway. Frontiers in bioscience : a journal and virtual library.

[32] Levy DE, Darnell JE (2002). Stats: transcriptional control and biological impact. Nature reviews Molecular cell biology.

[33] Vainchenker W, Constantinescu SN (2013). JAK/STAT signaling in hematological malignancies. Oncogene.

[34] Wu C, Guan Q, Wang Y, Zhao ZJ, Zhou GW (2003). SHP-1 suppresses cancer cell growth by promoting degradation of JAK kinases. Journal of cellular biochemistry.

[35] Carpenter RL, Lo HW (2014). STAT3 Target Genes Relevant to Human Cancers. Cancers.

[36] Huang X, Meng B, Iqbal J, Ding BB, Perry AM, Cao W, Smith LM, Bi C, Jiang C, Greiner TC, Weisenburger DD, Rimsza L, Rosenwald A, Ott G, Delabie J, Campo E (2013). Activation of the STAT3 signaling pathway is associated with poor survival in diffuse large B-cell lymphoma treated with R-CHOP. Journal of clinical oncology : official journal of the American Society of Clinical Oncology.

[37] Mascarenhas J, Hoffman R (2012). Ruxolitinib: the first FDA approved therapy for the treatment of myelofibrosis. Clinical cancer research : an official journal of the American Association for Cancer Research.

[38] Tamir I, Dal Porto JM, Cambier JC (2000). Cytoplasmic protein tyrosine phosphatases SHP-1 and SHP-2: regulators of B cell signal transduction. Current opinion in immunology.

[39] Jiao H, Berrada K, Yang W, Tabrizi M, Platanias LC, Yi T (1996). Direct association with and dephosphorylation of Jak2 kinase by the SH2-domain-containing protein tyrosine phosphatase SHP-1. Molecular and cellular biology.

[40] Chim CS, Fung TK, Cheung WC, Liang R, Kwong YL (2004). SOCS1 and SHP1 hypermethylation in multiple myeloma: implications for epigenetic activation of the Jak/STAT pathway. Blood.

[41] Pandey MK, Sung B, Ahn KS, Aggarwal BB (2009). Butein suppresses constitutive and inducible signal transducer and activator of transcription (STAT) 3 activation and STAT3-regulated gene products through the induction of a protein tyrosine phosphatase SHP-1. Molecular pharmacology.

[42] Mauldin IS, Tung KS, Lorenz UM (2012). The tyrosine phosphatase SHP-1 dampens murine Th17 development. Blood.

[43] Bittorf T, Seiler J, Zhang Z, Jaster R, Brock J (1999). SHP1 protein tyrosine phosphatase negatively modulates erythroid differentiation and suppression of apoptosis in J2E erythroleukemic cells. Biological chemistry.

[44] Paling NR, Welham MJ (2002). Role of the protein tyrosine phosphatase SHP-1 (Src homology phosphatase-1) in the regulation of interleukin-3-induced survival, proliferation and signalling. The Biochemical journal.

[45] Han Y, Amin HM, Franko B, Frantz C, Shi X, Lai R (2006). Loss of SHP1 enhances JAK3/STAT3 signaling and decreases proteosome degradation of JAK3 and NPM-ALK in ALK+ anaplastic large-cell lymphoma. Blood.

[46] Alicea-Velazquez NL, Jakoncic J, Boggon TJ (2013). Structure-guided studies of the SHP-1/JAK1 interaction provide new insights into phosphatase catalytic domain substrate recognition. Journal of structural biology.

[47] Bromberg JF, Wrzeszczynska MH, Devgan G, Zhao Y, Pestell RG, Albanese C, Darnell JE (1999). Stat3 as an oncogene. Cell.

[48] Catlett-Falcone R, Landowski TH, Oshiro MM, Turkson J, Levitzki A, Savino R, Ciliberto G, Moscinski L, Fernandez-Luna JL, Nunez G, Dalton WS, Jove R (1999). Constitutive activation of Stat3 signaling confers resistance to apoptosis in human U266 myeloma cells. Immunity.

[49] Yu H, Jove R (2004). The stats of cancer - New molecular targets come of age. Nature Reviews Cancer.

[50] Witzig TE, Price-Troska TL, Stenson MJ, Gupta M (2013). Lack of JAK2 activating non-synonymous mutations in diffuse large B-cell tumors: JAK2 deregulation still unexplained. Leuk Lymphoma.

[51] Demosthenous C, Han JJ, Stenson MJ, Maurer MJ, Wellik LE, Link B, Hege K, Dogan A, Sotomayor E, Witzig T, Gupta M (2015). Translation initiation complex eIF4F is a therapeutic target for dual mTOR kinase inhibitors in non-Hodgkin lymphoma. Oncotarget.

