# Supplementary material for: Loss of function mutations in PTPN6 promote STAT3 deregulation via JAK3 kinase in diffuse large B-cell lymphoma
Source: Oncotarget. 2015 Nov 9;6(42):44703–13. doi: 10.18632/oncotarget.6300 (PMC4792586; doi:10.18632/oncotarget.6300)
Supplement: Supplementary file 1 [file oncotarget-06-44703-s001.pdf]

Loss -of– function mutations in PTPN6 promote STAT3 deregulation *via* JAK3 kinase in diffuse large B-cell lymphoma

Supplementary Material

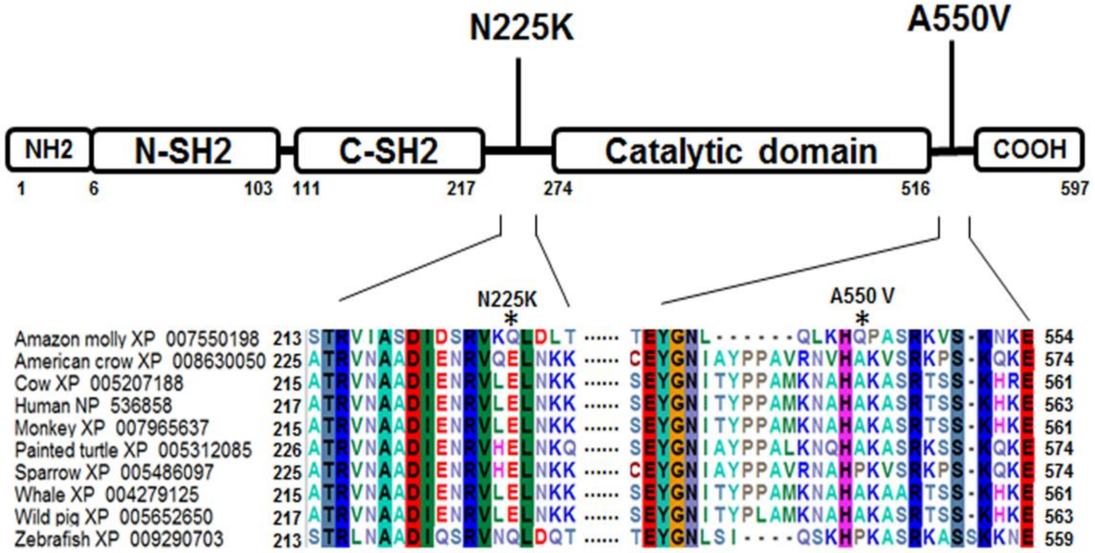

Supplemental figure 1: Modular structure of PTPN6 showing the N225K and A550V mutations. Alignment of part of PTPN6 protein sequence from various species shows that Asparagine 225 and Alanine 550 are non-conserved amino acids.
